# Sequence characterization and phylogenetic analysis of mitogenome of the *Acanthorhodeus chankaensis* Dybowsky from Cao’e River

**DOI:** 10.1080/23802359.2019.1710282

**Published:** 2020-01-14

**Authors:** Haifang Xu, Pengjie Yang, Wenjie Dai, Shoubao Yang

**Affiliations:** a College of Life Sciences, Shaoxing University, Shaoxing, Zhejiang, P. R. China;; b Department of Biology, Sanmen Experimental Junior High School, Taizhou, Zhejiang, P. R. China

**Keywords:** *Acanthorhodeus chankaensis* Dybowski, mitochondrial genome, characterization, phylogenetic analysis

## Abstract

In the present study, the complete mitogenome sequence of a *Acanthorhodeus chankaensis* Dybowsky from Cao’e River was sequenced and identified. The assembled mitogenome of *A. chankaensis* is 16,676** **bp in length, it contains 22 transfer-RNA genes, 13 protein-coding genes, 2 ribosomal-RNA genes, and 2 non-coding regions. It shows conserved gene arrangement with other Cyprinidae fishes. The overall nucleotide composition of *A. chankaensis* mitogenome sequence is A: 28.96%, G: 17.11%, T: 27.46%, and C: 26.47%. The phylogenetic analysis showed that the complete mitogenome could contribute to the phylogenetic analyses and population genetics study of *A. chankaensis* and *Acanthorhodeus* fish.

*Acanthorhodeus chankaensis* Dybowsky, a small-sized fish distributed widely in various water systems in China (Chen et al. 2005; Chen, Zhao, et al. 2013; Meng et al. 2016; Wang et al. 2019). It is part of the food web in natural waters because of its large population and plays an important role in maintaining water ecosystem balance. Except for its edible value, it also has ornamental value due to their marital color during the breeding season (Zhao et al. 2010; Wang et al. 2015, Wang 2019). However, *A. chankaensis* is suffering from rapid population reductions because of pesticide application, water pollution and increasing capture pressure (Wang et al. 2015). Cao’e River is the main channel in East China (Xie and Pan 2013; Han, 2018). Although *A. chankaensis* is a common fish in China, knowledge about it in Cao’e River is largely unknown.

The mitochondrial genome (mitogenome) sequence has compact gene arrangement, short coalescence time, and rapid evolutionary rate (Habib et al. 2012; Yang et al. 2018), which could provide useful data for phylogenetic analyses, population genetics and evolution study (Chen, Zhao, et al. 2013; Wang et al. 2015; Yang et al. 2015; Yu et al. 2019).

In the present study, a new mitochondrial genome of *A. chankaensis* (GenBank accession no. MN683735) was sequenced and annotated. *Acanthorhodeus chankaensis* was collected from Cao’e River, Zhejiang Province in China (30°00′08.4′′N, 120°52′49.7′′E) and kept in 99% ethanol in Shaoxing Aquatic Service Platform (SXAF191125). The total genomic DNA of *A. chankaensis* was isolated and was quantified by ultra-micro spectrophotometry. The PCR amplification was carried out using the following protocols: initial denaturation at 93 °C for 4** **min, followed by 38 cycles (94 °C for 45** **s, 50–52 °C for 35** **s, and 72 °C for 80** **s) and 1 final cycle of 6** **min at 72 °C.

The complete mitochondrial genome of *A. chankaensis* is 16,676** **bp in length, it contains a conserved arrangement with other Cyprinidae fishes (Wei et al. 2015; Yang et al. 2015), which include 22 transfer RNA genes, 13 protein-coding genes (PCDs), 2 ribosomal RNA genes, and 2 non-coding regions (control region and D-loop).

The nucleotide composition of *A. chankaensis* mitogenome sequence is A: 28.96%, G: 17.11%, T: 27.46%, and C: 26.47%. A similar A** **+** **T bias (56.42%) was seen with other vertebrate mitogenomes (Liu and Cui 2009; Wan et al. 2013; Pan et al. 2015).

The total sequence length of the PCD genes is 11,408** **bp. The total length of the tRNA genes is 1562** **bp, their sizes ranged from 68** **bp (*tRNA^Cys^*) to 76** **bp (*tRNA^Lys^* and *tRNA^Leu^*). The *12S* and *16S rRNA* genes are 958** **bp and 1679** **bp in length, respectively. Similar to other vertebrate mitogenomes, these two genes are located between the genes *tRNA^Phe^* and *tRNA^Leu(UUR)^* and are separated by the gene for *tRNA^Val^*.

The phylogenetic analysis showed that *A. chankaensis* was firstly clustered with another reported *A. chankaensis* (Lv et al. 2015), and then was clustered in the genus *Acanthorhodeus* with other *Acanthorhodeus* fishes (Figure 1). While it showed distant kinship with other Rhodeus fishes. This study provides useful data to phylogenetic analyses and population genetics of *Acanthorhodeus* fishes.

**Figure 1.**
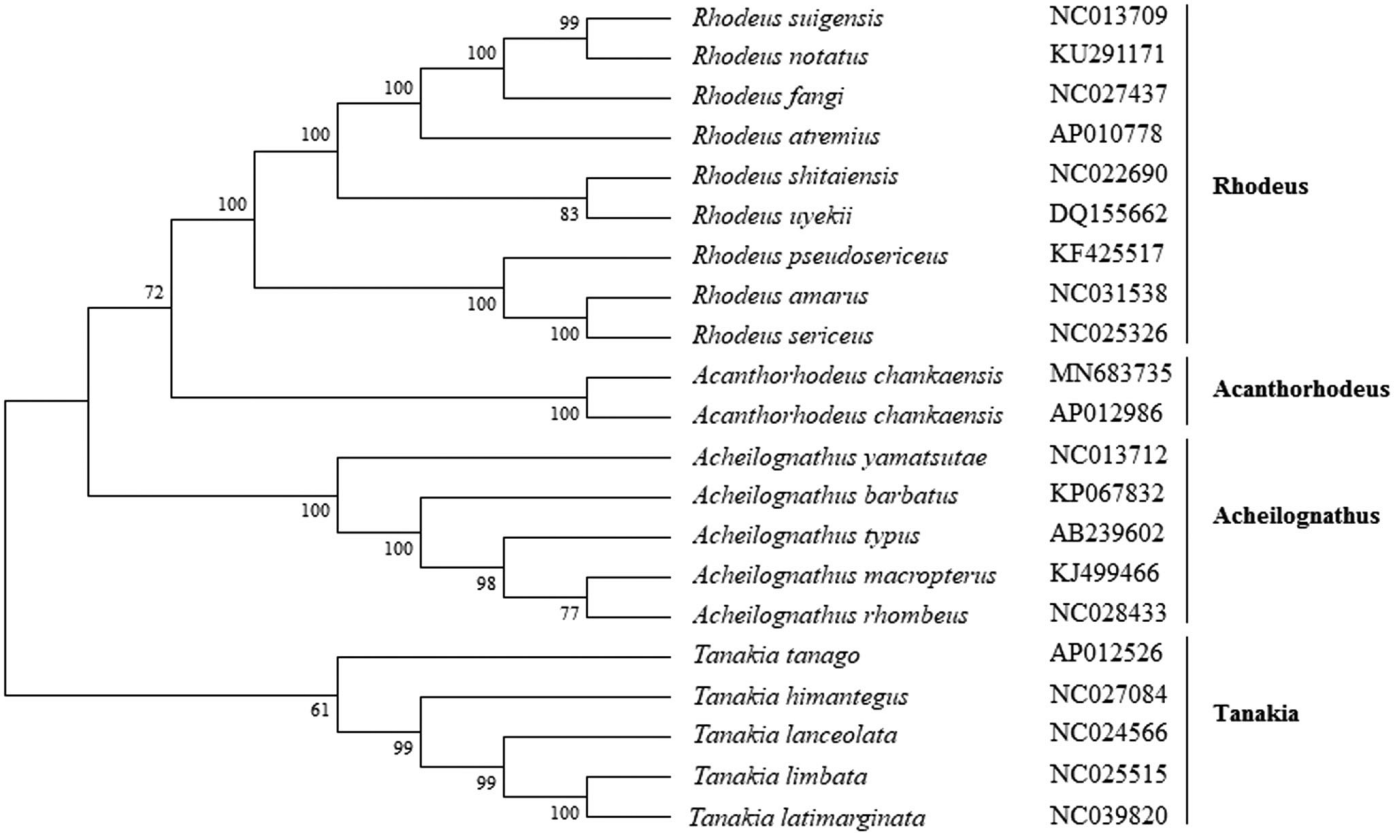
The phylogenetic analysis of *Acanthorhodeus chankaensis* and other Rhodeus fishes based on the mitogenome sequences.
